# A New Measure of Interpersonal Exploitativeness

**DOI:** 10.3389/fpsyg.2013.00299

**Published:** 2013-05-29

**Authors:** Amy B. Brunell, Mark S. Davis, Dan R. Schley, Abbey L. Eng, Manfred H.M. van Dulmen, Kelly L. Wester, Daniel J. Flannery

**Affiliations:** ^1^Department of Psychology, The Ohio State University at Mansfield, Mansfield, OH, USA; ^2^Criminal Justice Research Center, The Ohio State University, Columbus, OH, USA; ^3^Department of Psychology, The Ohio State University, Columbus, OH, USA; ^4^College of Public Health, Kent State University, Kent, OH, USA; ^5^Department of Psychology, Kent State University, Kent, OH, USA; ^6^Counseling and Educational Development, University of North Carolina at Greensboro, Greensboro, NC, USA; ^7^Mandel School of Applied Social Sciences, Case Western Reserve University, Cleveland, OH, USA

**Keywords:** exploitativeness, narcissism, measurement, reciprocity, social dilemma

## Abstract

Measures of exploitativeness evidence problems with validity and reliability. The present set of studies assessed a new measure [the Interpersonal Exploitativeness Scale (IES)] that defines exploitativeness in terms of reciprocity. In Studies 1 and 2, 33 items were administered to participants. Exploratory and Confirmatory Factor Analysis demonstrated that a single factor consisting of six items adequately assess interpersonal exploitativeness. Study 3 results revealed that the IES was positively associated with “normal” narcissism, pathological narcissism, psychological entitlement, and negative reciprocity and negatively correlated with positive reciprocity. In Study 4, participants competed in a commons dilemma. Those who scored higher on the IES were more likely to harvest a greater share of resources over time, even while controlling for other relevant variables, such as entitlement. Together, these studies show the IES to be a valid and reliable measure of interpersonal exploitativeness. The authors discuss the implications of these studies.

## Introduction

The social and behavioral sciences have long shown an interest in the concepts of exploitation and exploitativeness, or unfairly using others for profit or advantage. A June 2012 check of PsycINFO (1967-Present) revealed more than 7,700 records containing the term *exploitation*, with approximately 2,600 in the past 5 years alone. In addition, Sociology’s electronic library database, SocINDEX, contains more than 6,000 references related to exploitation; more than 900 of these records are from the past 5 years. The tendency to engage in exploitation – *exploitativeness* – has fewer citations, but nevertheless it is clear that social and behavioral scientists consider exploitation and exploitativeness important for theory, research and practice.

Across several disciplines in the social and behavioral sciences, recent work on reciprocity in social exchange indirectly raises questions about exploitativeness. The norm of reciprocity states that people treat others as they have been treated, repaying kindness with kindness and retaliating against those who inflict harm (Gouldner, 1960). Sociologists, including Coleman (1990) and Putnam (1993) consider reciprocity, along with the social networks in which people participate, to be a key component of social capital. Game theorists interested in cooperation routinely speak of exploitation as a common strategy for maximizing one’s own outcomes in an economic exchange. In addition, anthropologists (e.g., Yan, 2009) and criminologists (e.g., Wiebe, 2004), have either explicitly or implicitly attributed various aspects of social harmony and disharmony to the adherence to, or the departure from, the norm of reciprocity.

Much of the interest in exploitativeness has stemmed from its connection with narcissism. Psychoanalysts, including Kohut (1971) and Kernberg (1998), have argued that in addition to other traits, narcissists are interpersonally exploitative. These clinical perspectives have been codified in the Diagnostic and Statistical Manual (DSM-IV-TR; American Psychiatric Association, 2000), which refers to exploitativeness as a trait of those who suffer from narcissistic personality disorder.

From a personality-social psychology perspective, narcissism is an individual differences variable that can be assessed in normative samples (see Morf and Rhodewalt, 2001; Campbell et al., 2006; Campbell and Foster, 2007; for recent reviews). The Narcissistic Personality Inventory (NPI; Raskin and Terry, 1988), the most widely used measure of narcissism in normative samples (del Rosario and White, 2005; Brown et al., 2009), contains a facet that assesses exploitativeness, which reflects that exploitativeness is a feature of narcissism in the general population. However, we maintain that there are conceptual and empirical problems with exploitativeness as measured with the NPI. The interest scholars have in investigating interpersonal exploitativeness calls for the availability of a separate valid and reliable measure to employ in research. Therefore, our purpose was to: (1) discuss the definitional and empirical shortcomings of exploitativeness, as measured with the NPI; (2) propose a new definition of exploitativeness derived from theory on reciprocity and social exchange; (3) report preliminary data on a new measure of interpersonal exploitativeness; and (4) suggest some possible avenues for future research on interpersonal exploitativeness.

The Merriam-Webster Dictionary^1^(Accessed June 18, 2012) defines *exploitative* as “unfairly or cynically using another person or group for profit or advantage.” The suffix *-ness* conveys a state, condition, quality, or degree. We therefore can conclude that exploitativeness is “the state, condition, quality, or degree of unfairly or cynically using another person or group for profit or advantage.” Such standard dictionary definitions bespeak of violations of, or departures from, the norm of reciprocity.

However, when examining the NPI for the items that reflect exploitativeness (see Table 1), this notion of the violation of the norm of reciprocity appears to be missing. Irrespective of its psychometric properties, it could be argued that the Exploitativeness subscale has face validity issues. For example, Item no. 6, “I can usually talk my way out of anything,” does not suggest that the individual employing such a strategy will net a greater benefit than what is deserved. The same can be said for Item no. 16, “I can read people like a book.” It is highly conceivable that an individual could have such an ability without necessarily taking advantage of others. The fact that the NPI’s Exploitativeness items empirically hang together in a subscale (as assessed with factor analysis) does not necessarily mean they represent interpersonal exploitativeness. The term exploitativeness conveys the notion of taking unfair advantage of others. A perusal of the five pairs of items comprising the NPI’s Exploitativeness subscale reveals little that suggests exploitation. Rather, these items seem to tap such domains as manipulativeness and deceit, which may be related to interpersonal exploitativeness, but are not synonymous with it. Thus, the construct the exploitativeness subscale of the NPI is examining is unclear.

**Table 1:** **NPI exploitativeness subscale (Raskin and Terry, 1988) items**.

Item no	Narcissistic response
6	I can usually talk my way out of anything
13	I find it easy to manipulate others
16	I can read people like a book
23	Everybody likes to hear my stories
35	I can make anyone believe anything I want them to

In addition, the empirical data on the reliability of the NPI’s Exploitativeness subscale lend little support for its retention and use. The initial analyses by Raskin and Terry (1988) yielded a reliability coefficient of only 0.52. Subsequent studies employing the Exploitativeness subscale reported alphas at 0.60 or lower (e.g., Billingham et al., 1999; del Rosario and White, 2005; Reidy et al., 2008). This indicates that individuals do not respond reliably on the Exploitativeness subscale of the NPI, which serves as yet another justification for the need of a new and improved measure for assessing interpersonal exploitativeness.

We are not the first to comment on the lack of reliability and validity of the NPI’s dimensions. Campbell et al. (2004), for example, created a measure of psychological entitlement for the same reasons we have sought to create a new measure of exploitativeness. Reliable and valid measures that independently assess the facets of narcissism are needed to examine the predictive value of each construct. Furthermore, entitlement and exploitativeness, although separate aspects of narcissism, are also likely to be correlated. That is, when people are entitled, they are also likely to feel that it is acceptable to take advantage of others. Indeed, in previous work, Raskin and Terry (1988) reported a correlation of 0.29 between their Entitlement factor and their Exploitativeness factor, whereas Campbell et al. (2004) reported a correlation of 0.32 between the Psychological Entitlement Scale (PES) and NPI Exploitiveness. Consequently, we would expect a modest correlation between a new measure of exploitativeness and entitlement.

In view of the interest in exploitativeness and the problems detailed above, we designed a series of studies to develop and validate a new measure. It was our goal to create a brief measure for use along with other narcissism and narcissism-related measures. In Study 1, we created a pool of items to measure interpersonal exploitativeness and administered them to undergraduate subjects. In Study 2, we used factor analysis techniques to confirm the content and structure of the measure. In Study 3, we correlated the new scale with a number of other measures to assess convergent and discriminate validity. Finally, in Study 4, we tested the performance of the new scale in predicting behavior in a commons dilemma because behavior in this type of situation should determine if those who score higher on exploitativeness are more likely to exploit a common resource.

## Study 1

### Participants

Participants were 482 undergraduate students at a large Midwestern state university who completed the exploitativeness items as a part of mass testing in exchange for partial course credit. Nearly 90 percent of the students were Caucasian. Demographic and exploitativeness data were unavailable for 88 students, resulting in *N* = 394 (128 male, 257 female, 9 not reported) students, who were on average 19.37-years-old (SD = 3.11).

### Generation of the item pool

The intent was to create a pool of items which conveyed exploitativeness in the sense of violating the norm of reciprocity, that is, benefiting at the expense of others. We chose not to use items from the NPI or other existing measures that purportedly addressed exploitativeness. An initial pool of 33 items designed to measure interpersonal exploitativeness was independently generated by several of the authors and reviewed for clarity, vagueness, and redundancy by four subject matter experts whose specialties included clinical, social-personality, and quantitative psychology.

### Materials and procedure

Following informed consent, participants completed the 33-item version of the IES. We used a 7-point Likert response scale, with one indicating *strong disagreement* and seven indicating *strong agreement*. Scores across the 33 items were averaged such that a higher score was indicative of greater exploitativeness. For the present sample, α = 0.94, *M* = 2.84, SD = 0.92.

### Results and discussion

We examined the factor structure of the 33 items through exploratory factor analysis in SAS, using PROMAX, an oblique factor rotation method, although a similar exploratory factor analysis with VARIMAX, an orthogonal factor rotation method, produced qualitatively similar results. Nevertheless, examination of the screen plot indicated that one-factor should be retained, which accounted for 79.8% of the variance. The items, as well as their *M*, and SD are displayed in Table 2. Given our goal of creating a brief measure, items were retained if they loaded high (i.e., 0.70) and seemed to be related to the construct of exploitativeness, which resulted in a 6-item factor^2^. Cronbach’s α for the 6-item scale was 0.87. The average inter-item correlation = 0.54. Inter-item and corrected item-total correlations for these six items are displayed in Table 3.

**Table 2:** **Factor analysis and item means (SD) of the Interpersonal Exploitativeness Scale (Study 1)**.

Item no	Item	Factor loading	*M*	SD
1	What some people call taking advantage of others, I call taking care of myself	0.57	2.57	1.68
2	I believe in doing to others before they do unto me	0.37	3.57	1.93
3	I don’t take advantage of others. (reversed)	0.41	2.29	1.70
4	If I can get the upper hand, I will	0.36	4.33	1.68
**5**	**It doesn’t bother me to benefit at someone else’s expense**	**0.71**	**2.66**	**1.63**
6	I don’t mind taking advantage of someone else	0.68	2.10	1.43
7	It does not bother me if I get more of everything than others	0.63	3.03	1.72
8	I don’t like to use other people. (reversed)	0.41	2.56	1.91
**9**	**I’m perfectly willing to profit at the expense of others**	**0.72**	**2.48**	**1.56**
10	It’s just too bad if my gain is someone else’s loss	0.67	2.88	1.63
11	I’m far more concerned about my needs than the needs of others	0.61	2.91	1.67
**12**	**I’m less interested in fairness than getting what I want**	**0.70**	**2.56**	**1.42**
13	I feel I deserve more than others	0.60	2.56	1.67
**14**	**Vulnerable people are fair game**	**0.74**	**2.17**	**1.44**
15	People who let themselves be taken advantage of deserve it	0.63	2.84	1.72
16	Those that don’t get what they can while they can are saps/chumps	0.65	2.59	1.58
17	Offering to do things for others is good. (reversed)	0.39	1.96	1.33
18	Only fools fail to take what they want	0.67	2.91	1.61
19	It’s important to me everyone gets his or her fair share. (reversed)	0.40	2.87	1.51
20	All’s fair in love, war, and everything else	0.43	3.55	1.76
**21**	**Only weak people worry about fairness**	**0.71**	**2.25**	**1.46**
22	If it’s between me and another person, I will do whatever it takes to make sure my needs come first	0.60	2.68	1.60
23	I see nothing wrong with people taking what they want	0.62	2.86	1.59
24	The person who said “Every man for himself” was right	0.50	3.49	1.68
25	I say stick it to the other person before they stick it to you	0.64	2.86	1.56
26	I think it’s wrong to use others unfairly. (reversed)	0.43	2.53	1.74
27	I generally try to work a situation to my advantage	0.51	3.95	1.64
**28**	**Using other people doesn’t bother me very much**	**0.78**	**2.30**	**1.44**
29	I don’t like to impose on others. (reversed)	0.36	2.81	1.56
30	If I get more than others, so much the better	0.71	3.09	1.57
31	If I end up with more than others I feel guilty (reversed)	0.35	3.52	1.49
32	It feels good receiving more than I’m due	0.42	4.05	1.74
33	I prefer encounters that are fair (reversed)	0.53	2.35	1.40

Bolded items indicate the 6-item Interpersonal Exploitativeness Scale.

**Table 3:** **The Interpersonal Exploitativeness Scale: inter-item and corrected item-total correlations (Study 1)**.

Item		5	9	12	14	21	28	Corrected item-total correlations
5	It doesn’t bother me to benefit at someone else’s expense	–						0.66
9	I’m perfectly willing to profit at the expense of others	0.60	–					0.70
12	I’m less interested in fairness than getting what I want	0.53	0.49	–				0.65
14	Vulnerable people are fair game	0.53	0.55	0.52	–			0.69
21	Only weak people worry about fairness	0.44	0.56	0.49	0.57	–		0.66
28	Using other people doesn’t bother me very much	0.53	0.56	0.55	0.58	0.58	–	0.71

This study yielded a potential one-factor measure of interpersonal exploitativeness. In Study 2, we sought to confirm the structure of the 6-item IES that we found in Study 1.

## Study 2

### Participants

Participants were 535 (164 male, 354 female; 16 not reported) undergraduates at a large state university in the Midwest who provided informed consent and completed the IES in exchange for partial course credit. Nearly 94 percent of the participants were Caucasian, and the mean age was 19 years (SD = 3.17). For the present sample, α = 0.89, *M* = 2.40, SD = 1.21.

### Results and discussion

SAS PROC FACTOR was used to estimate a one-factor model for the 6-item IES using weighted least squares estimation. Chi-square for the model rejected the null hypothesis that the model perfectly fit the data (χ^2^ = 26.78, *df* = 9, *p* < 0.002). However, because the chi-square statistic is sensitive to sample size, we looked at other measures of fit as well (see Kaplan, 2000). The measure of comparative fit exceeds the 0.95 threshold that indicates an acceptable fit (CFI = 0.98; Mueller and Hancock, 2008). Moreover, the Standardized Root Mean Squared Residual is equal to 0.031, which meets the standard indicating good fit (SRMR < 0.08; Mueller and Hancock, 2008). Additionally, the Root Mean Square Error of Approximation is equal to 0.067, which is at the upper edge of the standard indicating good fit (RMSEA < 0.10 according to Browne and Cudeck, 1993; RMSEA < 0.06 according to Mueller and Hancock, 2008). With the 6-item scale confirmed, we next examined the convergent and discriminant validity of the IES by correlating it with other relevant constructs, including measures of narcissism.

## Study 3

### Participants

Participants were 228 Introductory Psychology students (112 male; 116 female) who participated in exchange for partial course credit. On average, participants were 19.35 years old (SD = 2.78). About 78.9% of the participants self-identified as Caucasian; another 11.4% self-identified as African-American.

### Materials and procedure

Following informed consent, participants were asked to complete a series of measures to demonstrate the construct validity of the IES (for the present sample, α = 0.89, *M* = 2.53, SD = 1.38 for the IES). We selected several measures which theoretically should correlate with interpersonal exploitativeness. First, we chose Raskin and Terry’s (1988) original Exploitativeness subscale of the NPI, in addition to the Entitlement/Exploitativeness subscale that Ackerman et al. (2011) recently reported in a paper reassessing the factor structure of the NPI. Our criticisms of the NPI’s Exploitativeness subscale notwithstanding, exploitativeness has been identified as part of narcissism’s maladaptive side. As such, we predicted that it would be positively correlated with the IES inasmuch as interpersonal exploitativeness is also considered socially maladaptive. Thus, participants completed the 40-item version of the NPI (Raskin and Terry, 1988). Participants were asked to choose between a pair of statements (e.g., “I can usually talk my way out of anything.” or “I try to accept the consequence of my behavior.”). The non-narcissistic response is assigned a score of 0 and the narcissistic response is assigned a score of 1. Total NPI scores were computed by summing responses to all 40 items, with higher scores reflecting higher levels of narcissism. NPI Exploitativeness scores (Raskin and Terry, 1988) and Entitlement/Exploitativeness scores (Ackerman et al., 2011) were computed by summing the scores across the respective subscale items. For our sample, α = 0.84, *M* = 16.30, SD = 6.75 for total NPI scores, α = 0.48, *M* = 1.88, SD = 1.35 for the Raskin and Terry (1988) NPI Exploitativeness subscale, and α = 0.39, *M* = 0.94, SD = 0.99 for the Ackerman et al. (2011) Entitlement/Exploitativeness subscale.

Second, we selected the Pathological Narcissism Inventory (PNI; Pincus et al., 2009) which is a 52-item multidimensional measure of pathological narcissism. The dimensions include the assessment of contingent self-esteem, exploitativeness, self-sacrificing self-enhancement, hiding the self, grandiose fantasy, devaluing, and entitlement rage. Respondents use 6-point scales to indicate the extent to which each item is “like me” (0 = *not at all like me* and 5 = *very much like me*). For our purposes, we report correlations with PNI scores, which were averaged across the 52 items, as well as the two most relevant subscales for our purposes: the 5-item Exploitative subscale (e.g., “I find it easy to manipulate people.”) and the 8-item Entitlement Rage (e.g., “I get mad when people don’t notice all that I do for them.”). It should be noted that the items assessing exploitativeness on the PNI are adapted from the exploitativeness subscale of the NPI. We expected that the IES would correlate positively with the PNI and its dimensions of exploitativeness and entitlement rage. For our sample, α = 0.94, *M* = 2.51, SD = 0.69 for PNI scores, α = 0.77, *M* = 2.61, SD = 1.02 for the PNI Exploitative subscale, and α = 0.85, *M* = 2.28, SD = 1.00 for the PNI Entitlement Rage subscale.

Next we included the 9-item PES (Campbell et al., 2004), which was developed to assess the extent to which one feels he or she is entitled to more than others or deserves more than others. This measure was developed because the NPI’s Entitlement subscale lacks adequate reliability and validity to assess entitlement. A sample item is “I feel entitled to more of everything.” Respondents use 7-point scales to indicate the extent to which they agree with each statement (1 = *strong disagreement*, 7 = *strong agreement*). Given that we believe that exploitativeness and entitlement go hand in hand, we expected a positive correlation between these two dimensions. For our sample, α = 0.86, *M* = 3.32, SD = 1.20.

We also included the 10-item Rosenberg Self-Esteem Scale (Rosenberg, 1965), a commonly used measure of global self-esteem, because a positive correlation between the NPI and self-esteem is commonly reported (e.g., Campbell et al., 2002, 2007). It might be the case that those who score high on self-esteem might be more likely to exploit others. However, given the more communal nature of those with high self-esteem compared to those who score high on narcissism (Campbell et al., 2002), we did not expect those who score high on self-esteem to be more exploitative. A sample item from the Rosenberg Self-Esteem Scale is “I feel that I have a number of good qualities.” Respondents use 5-point scales to rate the extent to which they agree with each statement (1 = *strong disagreement*, 5 = *strong agreement*). For the present sample, α = 0.84, *M* = 3.35, SD = 0.65.

Lastly, we selected the Positive Reciprocity and Negative Reciprocity subscales of the Personal Norm of Reciprocity Questionnaire (Perugini et al., 2003). Positive reciprocity connotes a preference for behaving fairly in interpersonal exchanges. The Positive Reciprocity subscale contains items such as “When someone does me a favor, I feel committed to repay him/her.” and “If someone asks me politely for information, I’m really happy to help him/her.” Negative Reciprocity conveys a preference for retaliating against those who are responsible for unfair treatment. A sample item is “If I suffer a serious wrong, I will take my revenge as soon as possible, no matter what the costs.” Responses were measured using a 7-point scale ranging from *not true for me* to *very true for me*. We hypothesized that the IES would correlate negatively with positive reciprocity inasmuch as those who are interpersonally exploitative should be at ease taking favors from others without feeling obligated to repay them. We hypothesized that the IES would correlate positively with negative reciprocity inasmuch as retaliatory behavior has been associated with the maladaptive traits of narcissism. For our sample, Positive Reciprocity: α = 0.86, *M* = 5.83, SD = 0.89; Negative Reciprocity: α = 0.90, *M* = 3.83, SD = 1.40.

### Results and discussion

Correlations between the IES and the other measures of narcissism, self-esteem, entitlement, and reciprocity are displayed in Table 4. As would be expected, the IES was positively associated with other measures of narcissism, such as the NPI, the PNI, as well as their respective subscales assessing exploitativeness and entitlement. The IES was also correlated with the PES. The IES was not associated with the Rosenberg Self-Esteem Scale, suggesting that one’s own feelings of adequacy or inadequacy is not prerequisite for exploiting others.

**Table 4:** **External correlations between IES factors and validity measures (Study 3)**.

Measure	*r*
NPI total score	0.35***
NPI Exploitativeness	0.40***
Ackerman et al. Entitlement/Exploitiveness	0.40***
Pathological Narcissism Inventory (PNI)	0.26***
PNI Exploitative	0.44***
PNI Entitlement Rage	0.36***
Psychological Entitlement	0.40***
Rosenberg Self-Esteem	−0.03
Positive Reciprocity	−0.25***
Negative Reciprocity	0.48***

***p < 0.001.

Just as noteworthy were the significant associations between the IES and both positive and negative reciprocity. Positive reciprocity conveys the tendency to behave fairly in interpersonal exchanges (Perugini et al., 2003). The negative correlation between the IES and positive reciprocity indicates that those who score higher on the IES are less likely to engage in fair exchanges. The positive correlation between the IES and negative reciprocity reveals that exploitative people have an individual preference for retaliatory behavior in response to real or perceived wrongs. Taken together, Studies 1–3 suggest that the IES is an internally consistent and valid measure of interpersonal exploitativeness. Our next step was to examine its performance in a social dilemma.

## Study 4

In the commons dilemma, participants have the opportunity to harvest common resources either selfishly or more judiciously for the common good (Hardin, 1968). That is, individuals can either selfishly harvest more for themselves, which depletes the available resources more quickly at the expense of others, or they can consider the needs of the community and harvest resources more responsibly, which ultimately benefits all concerned. We consider the commons dilemma particularly relevant to the notion of exploitativeness inasmuch as selfish harvesting behavior could be indicative of a personality trait.

For example, Campbell et al. (2005) used a commons dilemma involving forestry companies to test the relationship between narcissism and selfish behavior. They theorized that those who are more narcissistic are more likely to harvest greater amounts of forest, irrespective of the long-term consequences for the limited community resource. Using the NPI as their measure of narcissism, they confirmed their hypothesis, finding that higher NPI scores do predict exploitation of the forest.

Campbell et al. (2005) suggest that narcissists’ exploitative nature is at the core of their selfish behavior. However, Campbell et al. used NPI total scores to examine behavior in the commons dilemma and did not report which specific facet of narcissism was driving the behavior. However, in a separate study, Campbell et al. (2004) found that individuals who scored higher on psychological entitlement (a) reported more greed and (b) would desire to cut more forest on the first trial; however, this latter finding was not replicated (Campbell et al., 2005). Thus, we sought to address whether exploitativeness or entitlement predicts performance in the commons dilemma. To the extent that it is exploitativeness, our data would support Campbell et al. (2005) claim about narcissism.

The purpose of Study 4, then, was to test the relationship between scores on the IES and both short- and long-term behavior in the commons dilemma. The benefit of conducting a study on the commons dilemma was that it enabled us to examine the performance of the IES with respect to actual behavior. Also in Study 4, we were able to examine the unique role of the IES above and beyond the role of entitlement. We expected that those who scored higher on exploitativeness would readily take advantage of others and exploit the common resource at a greater rate than those who scored lower on exploitativeness. That is, to the extent that an individual is exploitative, he or she should be willing to exploit others for their own gain.

### Participants

Participants were 208 (79 male, 129 female) undergraduate students at a regional campus of a state university in the Midwest, who participated in exchange for partial course credit (*M*_age_ = 19.39, SD = 3.24). Most of the sample (78.8%) self-identified as Caucasian and 11.1% self-identified as African-American.

### Materials and procedure

Following informed consent, participants first completed the 6-item Interpersonal Exploitativeness Scale (IES; α = 0.86, *M* = 2.29, SD = 1.10) and the 9-item PES (Campbell et al., 2004; α = 0.84, *M* = 3.32, SD = 1.09). They then engaged in a competitive task in dyads, which was based on previous research about the tragedy of the commons (Sheldon and McGregor, 2000; Campbell et al., 2005). The researcher gave participants a booklet that contained the explanation of the commons dilemma. Specifically, participants were told that they represented a forestry company and that their individual goal was to harvest as much forest as possible. They were also told (a) there was another company harvesting the forest at the same time, (b) they could choose to harvest 0–10 ha of forest per year, and (c) there were only 100 ha of forest, which regrew by 10% after each annual harvest.

Participants then used 7-point scales (1 = *not at all* and 7 = *a great deal*) to answer two questions. First, they were asked to indicate their acquisitiveness by rating the extent to which they wanted to profit more than the other company (*M* = 5.71, SD = 1.17). Second, participants were asked to indicate their apprehension by rating the extent to which they thought that the other company wanted to profit more than they did (*M* = 6.22, SD = 1.11). After answering these two questions, participants began the bidding process. Each participant privately indicated the number of hectares that they wanted to harvest on a “bid sheet.” The researcher then (a) added together the total number of hectares harvested in the round, (b) subtracted that value from the amount of forest available (which started at 100 ha), (c) added 10% to this value, and (d) announced the total amount of hectares available for the next round. For example, if in the first round the combined harvest was 10 ha, the researcher would subtract 10 from 100, and then add in 10% (9 ha). The researcher would then announce that there was 99 ha of forest for the next round. This process was repeated up to 20 rounds (which was not revealed to participants ahead of time) or until the harvest was completely exhausted. Participants were then debriefed and thanked for their time.

### Results and discussion

As anticipated, the IES and the PES were correlated, *r* = 0.39, *p* < 0.001. The IES was negatively correlated with apprehension (*r* = −0.16, *p* < 0.05) but not with acquisitiveness (*r* = 0.10, *p* = 0.14) whereas the PES was correlated with acquisitiveness (*r* = 0.25, *p* < 0.001) but not apprehension (*r* = −0.13, *p* = 0.06). Acquisitiveness and apprehension were also positively correlated (*r* = 0.43, *p* < 0.001). When the IES was entered into a regression equation predicting acquisitiveness while controlling for apprehension, it significantly predicted acquisitiveness (β = 0.18, *t* = 2.90, *p* < 0.01). However, when the PES was also added to the equation, the PES was significant (β = 0.28, *t* = 4.33, *p* < 0.001) whereas the IES was not (β = 0.08, *t*(202) = 1.19, *p* = 0.24). Next, the IES was entered into a regression equation predicting apprehension while controlling for acquisitiveness. The IES significantly predicted lower apprehension (β = −0.21, *t*(202) = −3.38, *p* < 0.001), and remained significant (β = −0.14, *t* = −2.08, *p* < 0.05), even while controlling for the PES (β = −0.20,*t*(202) = −2.99, *p* < 0.01). When added to the models, gender was not a significant predictor of acquisitiveness (β = 0.04, *t*(201) = 0.64, *p* = 0.52) or apprehension β = −0.06, *t*(201) = −0.99, *p* = 0.32). Thus, it appears that the PES was more strongly associated with acquisitiveness than was the IES. Both the PES and the IES were associated with lower apprehension, findings which are supported by previous research that found that narcissists experience less apprehension than non-narcissists when engaging in competitive tasks (Morf et al., 2000).

The mean amount harvested was 82.81 (SD = 22.17) hectares; the mean number of rounds the dyads completed was 13.18 (SD = 3.96) rounds. We investigated the influences of gender and exploitativeness on total harvested and on the total number of rounds using multiple regression. Because there was no difference in total harvested and total number of rounds within dyads, we constructed the regression models with dyads rather than individuals (i.e., using individuals as opposed to dyads would have inflated sample size). We did not find a significant influence of the dyad’s gender or exploitativeness composition on the total amount harvested (β_Exploit_ = 0.81, *t*(101) = 0.55, *p* = 0.58, β_Gender_ = −4.64, *t*(101) = 1.44, *p* = 0.15) and the total number of rounds (β_Exploit_ = 0.06, *t*(101) = 0.22, *p* = 0.83, β_Gender_ = −0.36, *t*(101) = 0.62, *p* = 0.54).

To investigate the role of exploitativeness on allocation behavior in each round, we constructed a growth curve model for indistinguishable dyads by employing a multilevel model (MLM). Such a model allowed for the construction of an MLM version of an Actor-Partner Interdependence Model (APIM; Cook and Kenny, 2005). APIMs allow for the simultaneous estimation of the influence of an individual’s predisposition on their own behavior and their partner’s behavior; dyad members were indistinguishable. Our model was procedurally comparable to the model outlined by Kashy et al. (2008). We specify our model below:

$$\begin{array}{llll} Y_{\mathrm{ijk}}= & \;\beta_{\text{0}\mathrm{ij}}+\beta_{\text{01}}{\text{ActIES}}_{\mathrm{ij}}+\beta_{\text{02}}{\text{PartIES}}_{\mathrm{ij}}+\beta_{\text{03}}{\text{Totalrounds}}_{\mathrm{ij}}\; & & \;\\ & +\beta_{\text{04}}{\text{Gender}}_{\mathrm{ij}}+\beta_{\text{1}\mathrm{ij}}{\text{Round}}_{\mathrm{ijk}}+\beta_{\text{11}\mathrm{ij}}\text{Round}\times {\text{ActIES}}_{\mathrm{ijk}}\; & & \;\\ & +\beta_{\text{12}\mathrm{ij}}\text{Round }\times \;{\text{PartIES}}_{\mathrm{ijk}}\;+\;\beta_{\text{13}}\text{Round }\times \;{\text{Gender}}_{\mathrm{ijk}}+e_{\mathrm{ijk}}\; & & \; \end{array}$$

In the specified equation, *i* corresponds to the individual, *j* specifies the dyad, and *k* indicates the round. Because variables were mean-centered, the intercept β_0*ij*_ was not theoretically relevant. Level-1 variables (i.e., round) were centered within the dyad, such that 0 represents the study midpoint for any given dyad (i.e., a dyad that completed 20 rounds would have a midpoint at round 10, but a dyad that completed 8 rounds would have a midpoint at round 4). The variable *Gender* was a dichotomous variable with male = 0 and female = 1. As per the recommendation of Kashy et al. (2008), we created dummy codes to represent the actor and partner roles within the random effects portion, as to correctly specify the model. Furthermore, *Act*IES and *Part*IES specify mean IES scores for the actor and partner, respectively. In line with Kashy et al. (2008), we allowed level-2 intercepts and slopes to covary both within and across dyad members. Thus the following level-2 relationships were specified as follows:

$$\begin{array}{cc} \beta_{0\mathrm{ij}}=\gamma_{0\mathrm{ij}}+u_{0\mathrm{ij}} & \\ \beta_{1\mathrm{ij}}=\gamma_{1\mathrm{ij}}+u_{1\mathrm{ij}} & \end{array}$$

Because participants were randomly paired, we constrained the covariance structure such that there was only one intercept variance, slope variance, intercept covariance, slope covariance, within-person intercept-slope covariance, and between-person intercept-slope covariance (see Kashy et al., 2008 web appendix for further details on this covariance matrix specification). We modeled the residual variances and covariances by employing compound symmetry, such that residuals were constrained to be equal across rounds and equal between members of each dyad for each round. We ran a comparable model where we relaxed the assumption of equality across rounds and found negligible differences in fit and estimates. In addition, we specified Satterthwaite corrections for denominator degrees of freedom, as recommended by Kashy et al.(2008), though other specifications produced nearly identical results (e.g., dividing degrees of freedom into between-participant and within-participant portions). Fixed-effects coefficients, standard errors, and statistical tests are presented in Table 5; random-effect coefficients, standard errors, and statistical tests are presented in Table 6.

**Table 5:** **Fixed-effect solutions from the growth curve model (Study 4)**.

Variable	Coefficient	Standard Error	*t*-value
Intercept	0.243	0.037	6.53***
Round	0.039	0.019	2.10*
*Act*IES	0.028	0.048	0.57
*Part*IES	0.075	0.057	1.32
Gender	0.060	0.092	0.65
Totalrounds	−0.219	0.007	−30.29***
Round × actIES	0.037	0.016	2.26*
Round × partIES	0.048	0.018	2.74**
Round × gender	0.082	0.034	2.45*

*p < 0.05, **p < 0.01, ***p < 0.001.

**Table 6:** **Random-effect solutions from the growth curve model (Study 4)**.

Covariance parameter	Coefficient	Standard error	*z*-value
LIN(1)	1.51	0.238	6.35***
LIN(2)	−1.56	0.238	−6.57***
LIN(3)	0.03	0.006	6.06***
LIN(4)	0.01	0.006	2.12*
LIN(5)	0.06	0.021	2.71**
LIN(6)	0.01	0.021	0.55
Compound symmetry	0.11	0.11	0.97
Residual	3.86	0.16	24.22***

LIN(1) represents the significant estimated parameter of the variance of the intercepts and slopes. LIN(2) represents the significant estimated covariance of the intercepts. LIN(3) and LIN(4) represent the significant variance of the slopes for the dyad members. LIN(5) refers to the significant within-participant intercept-slope covariance, whereas LIN(6) represents non-significant differences in the between-participants intercept-slope covariance. Results indicated a non-significant coefficient for the estimate of the covariance of compound symmetry. Lastly, the significant residual variance indicates that there was substantial variation beyond what was explained by the level-1 component within the model (i.e., round number).*p < 0.05, **p < 0.01, ***p < 0.001.

Results of the growth curve model indicate a significant effect of round number, the average *b* = 0.04, *t*(104) = 2.08, *p* = 0.04. In particular, participants were more likely to allocate more for themselves in later rounds than in earlier rounds. In a related model we included a quadratic term for round number to test whether participants allocated more in early and later rounds than in middle rounds, the estimate of this parameter was non-significant, *b* = 0.004, *p* = 0.086. Consistent with the nature of the commons dilemma, dyads that played more rounds allocated less on average per round (i.e., developed mutual cooperation and thus sustained the game longer), *b* = −0.22, *t*(131) = −30.29, *p* < 0.0001.

We next investigated the role of gender differences in allocation behavior. Results indicated that women did not allocate significantly more than men at the midpoint of the study, *b* = 0.06, *p* = 0.51. In contrast, women appeared to allocate more in later rounds, compared to earlier rounds, than did men, *b* = 0.08, *t*(157) = 2.45, *p* = 0.02.

To investigate the role of actor effects (i.e., the influence of individual *i*’s IES score on their own allocation behavior) and partner effects (i.e., the influence of individual *i*’s IES score on their partner’s allocation behavior), we created variables *Act*IES and *Part*IES, as per the recommendations of Kashy et al. (2008). Results indicated no overall influence of an individual’s exploitativeness, *b* = 0.03, *p* = 0.56 nor their partner’s exploitativeness on their allocation behavior, *b* = 0.08, *p* = 0.18, at the midpoint of the study. In contrast, higher exploitativeness was associated with larger allocations as the study progressed, both for the actor’s exploitativeness, *b* = 0.04, *t*(164) = 2.26, *p* = 0.03, and the partner’s exploitativeness, *b* = 0.05, *t*(201) = 2.74, *p* = 0.007. That is, both the actor’s and their partner’s exploitativeness was positively related to increases in allocations in later rounds. As there was a null influence of exploitativeness on allocation decisions at the midpoint of the study, the significant interactions indicate that higher exploitativeness was associated with lower allocations (i.e., more cooperation) at the beginning of the study and higher allocations (i.e., less cooperation) at the end of the study. Testing an interaction of exploitativeness with round and gender (i.e., if gender influenced the relationship between either the actor or partner’s exploitativeness, round, and allocation behavior) produced null results, *p* = 0.91 and *p* = 0.18, respectively.

One consideration is whether the observed effects are uniquely a product of exploitativeness, or whether they could be similarly predicted by a related construct. We tested this consideration by adding the PES to the MLM. When including both the IES and the PES (i.e., both actor and partner effects for PES) in the model, neither *Act*PES, *b* = −0.01, *p* = 0.92, nor *Par*PES, *b* = 0.02, *p* = 0.65 influenced overall allocation behavior at the midpoint of the study. Furthermore, neither *Act*PES, *b* = −0.03, *p* = 0.10, nor *Part*PES, *b* = −0.02, *p* = 0.39, interacted with round^3^. With PES included within the MLM, both the *Act*IES × Round interaction *b* = 0.06, *t*(180) = 2.56, *p* = 0.01, and the *Part*IES × Round interaction remained significant, *b* = 0.05, *t*(205) = 1.94, *p* = 0.05. Taken together, the current results provide evidence in support of the predictive validity of the IES scale in terms of allocation behavior.

This study was designed to assess the role of interpersonal exploitativeness in a commons dilemma in which resources could be used irresponsibly or conserved. We hypothesized that those who scored higher on the IES would be less likely to conserve common resources. Results suggested that higher IES scores are related to cooperative behavior at the beginning of the study and less cooperative behavior (i.e., less conservation of common resources) at the end of the study. Perhaps interpersonally exploitative people use this strategy to earn the partner’s trust early on and then defect later on to “win.” This pattern of findings remained significant even when controlling for other relevant constructs, such as psychological entitlement, which did not influence allocation behavior in the commons dilemma.

## General Discussion

The purpose of these studies was to develop and validate a new measure of interpersonal exploitativeness grounded in norms of reciprocity and exchange. In Study 1, the initial pool of 33 items was administered to a sample of undergraduate students who participated in mass testing. Exploratory factor analysis yielded one-factor that consisted of six items, with high internal reliability. Confirmatory factor analysis in Study 2 confirmed the fit of a one-factor solution consisting of the six items. The single factor solution evidenced satisfactory psychometric properties.

In Study 3, the IES was correlated with other measures to determine the extent to which the IES was associated with narcissism and reciprocity. As predicted, the IES was positively associated with narcissism, narcissistic exploitativeness, entitlement, and negative reciprocity. Also as predicted, the IES was negatively associated with positive reciprocity. The associations between the IES and the validation measures were in the expected directions.

Finally, in Study 4, we employed a commons dilemma to assess how interpersonally exploitative individuals behaved when confronted with a series of resource management decisions. Those who scored higher on the IES were initially cooperative but then became increasingly less cooperative over time in response to the partner’s behavior. This behavior led to greater resource destruction of the commons, which in turn prevented more exploitative people from harvesting more overall.

### Implications

Despite the widespread use of the NPI over the past 30 years, a number of social-personality psychologists have expressed dissatisfaction with it (Brown et al., 2009). Indeed, the shortcomings of the NPI have prompted the development of a number of new measures to assess narcissistic traits such as entitlement (Campbell et al., 2004) and grandiosity (Rosenthal et al., unpublished). The IES can be used to assess the exploitativeness trait associated with narcissism. We agree with Brown et al. (2009) that it is difficult, if not impossible, to examine the complexities of narcissism with a single, albeit multidimensional measure.

We also think that the IES has uses outside the context of narcissism. For example, it has been argued that the personality traits that comprise the Dark Triad – Machiavellianism, narcissism, and psychopathy – share features including an exploitative interpersonal style (Palmen et al., 2011). The Dark Triad has been associated with a variety of socially maladaptive behaviors. Despite the strong connection between narcissism and interpersonal exploitativeness, investigators should be open to the possibility that the latter trait may be evident in other personality constellations such as the Dark Triad, and future research should employ the IES to explore its possible role.

Likewise, future research should examine how the IES corresponds to other personality measures that have facets that examine violations from the norm of reciprocity. For example, the HEXACO Personality Inventory (Lee and Ashton, 2004) has a domain for honesty-humility that includes subscales that examine fairness, modesty, and greed avoidance, and recent investigation of the Social Exchange Styles Questionnaire included subscales that assess fairness, benefit-seeking, and overinvestment (Leybman et al., 2011).

There are a few of limitations of our studies to mention. First, in the present set of studies, we did not assess test-retest reliability, which future research should address. Also, as with any research that employs college student subjects with restricted demographic characteristics, our ability to infer to the general population might be limited. And although the findings from the commons dilemma suggest that those who score higher on the IES are more likely to exploit resources, it remains unclear how these individuals would behave in their daily lives outside a laboratory setting. Despite such limitations, the IES should serve as a useful measure for those interested in interpersonal exploitativeness as either a stable trait or a *modus operandi*.

It is also important to note that the consequences of higher interpersonal exploitativeness might not always be negative. For example, in Study 4, participants were told that their individual goal was to harvest as much forest as possible. Those who scored higher on the IES cooperated early on and then defected at a later point in time, possibly to keep their opponents from outperforming them on this goal. In a “dog-eat-dog” world, a certain level of interpersonal exploitativeness might be beneficial to the individual striving for success, such as climbing the corporate ladder. Future research is needed to examine the beneficial and detrimental consequences of interpersonal exploitativeness.

## Conclusion

The IES was developed to provide scholars with a reliable and valid measure of interpersonal exploitativeness. Across four studies, the IES was demonstrated to be a reliable and valid measure of exploitativeness. We believe that the IES will be useful for examining the role of exploitativeness in understanding a wide range of social behaviors.

## Conflict of Interest Statement

The authors declare that the research was conducted in the absence of any commercial or financial relationships that could be construed as a potential conflict of interest.

## Footnotes

^1^http://merriam-webster.com

^2^Item 30 was not administered in Studies 3 and 4 and was therefore eliminated from further analysis.

^3^Even when removing the actor and partner effects of IES from the MLM, PES failed to attain statistical significance in any main effect or interaction for either actor or partner.
